# Annotations

**Published:** 1893-01-28

**Authors:** 


### ANNOTATIONS.

**The "Bromide Habit."** THE use of bromide of potassium, under the mutilated name of "Bromide," is almost as common in our generation as the use of "blood-letting" was in the first half of the century. Dr. Samuel Wilks, whom we all sincerely respect, is ashamed of this state of things; and if he is ashamed of it, all the rest of us ought to be. Dr. Wilks has written an original article for the January number of the *Practitioner*—an article whose originality and plainness of speech are exceedingly refreshing in these times of medical pomposity and padding. Dr. Wilks actually discusses medical subjects with unvarnished simplicity. But then he is not young. On the subject of "Bromide" he has the temerity to be even amusing. "Let me say," he writes, "as regards the latter drug (bromide) that a medicine of so universal application cannot be of much worth." Rash Dr. Wilks! "I know of no disease in which I have not seen it given. . . . It cannot be said that it has ever cured a disease. . . . I have never read the prescriptions of certain obstetric physicians without observing that bromide of potassium is an ingredient." Weak women make wily physicians. Dr. Wilks despises the former and hates the latter, as we all ought to do. "It is a curious, but not a very exhilarating exercise," he says, "to look over one's note-books and observe the prime cause of the trouble for which bromide was prescribed." . . . "Worry in business—bromide cf potassium; loss on the Stock Exchange—bromide of potassium; quarrel with the cook—bromide of potassium; loss of a pet dog—bromide of potassium; blighted affections—bromide of potassium; irritable stepmother—bromide of potassium; and so on." This is the state of things at the present day and hour gentlemen of the sage look and the crabbed hieroglyphics! It does not say much for the "progress of science" among those whose practice deals chiefly with the more sensitive sex. "Bromide" is to the "ladies'" doctor what chocolate creams are to the weak-minded governess—a means of quieting the "emotions" for a more or less brief period. The chocolate creams in due time derange the liver and destroy the digestion. The bromide also, if persisted in for even a moderate period, impairs the nervous tone and permanently destroys the happiness and mental capacity of the patient. All this may be grist to the ladies doctors' mill, but it is destruction to the lady. "The bromide habit" seldom produces such hopeless wreck of mind and character as the chloral or morphia habit; but where one person is injured by chloral a hundred suffer from the enervating and misery-producing effects of bromide. Good in its place, it is a most pernicious drug for prolonged and indiscriminate use; and even when used by order of the physician, we have it on the authority of Dr. Wilks that discrimination is too often conspicuous by its absence.

**Soft v. Hard Water.** ALL sensible people are thankful when they can get hold of knowledge which can be used in practice. Our constant contention is that knowledge can hardly be called knowledge at all until it has been tested by experiment and practice. Everybody likes soft water, for example, but many half-scientific people have a kind of idea that hard water, that is water with carbonate of lime dissolved in it, may be of some value in the nutrition and development of bones, and especially in the development of children's bones. "Nothing of the kind," says in effect Dr. J. M. Fox, in a paper addressed to the Sandbach Local Board of Health. Dr. Fox informs us that practical information on these important questions is not easily available, and that we can all confirm. He himself has worked out the subject, and has strengthened his own convictions by the experience of eminent chemists, and the evidence given before a Royal Commission appointed *ad hoc*. The principal use of water in the human body is for solvent purposes, argues Dr. Fox. But if that be so, it is manifest that water which has 70, or 80, or even 100 grains of solid matter per gallon dissolved in it must be much less powerfully solvent than water which has not more than five or ten grains. The water which is used up in dissolving the lime cannot dissolve other soluble substances—at any rate, not to the full extent of the natural solvent power of unadulterated water. It is sometimes argued, as we have said, that water having lime dissolved in it may, when drunk, give up its lime to the body, and so help in the formation of bones. On this point Dr. Fox quotes Sir Lyon, now Lord Playfair. Sir Lyon Playfair, as he then was, on being questioned by the Royal Commission, replied as follows: "I have seen evidence given in cases of water supply, not only that it was desirable for health, but that it (carbonate of lime) was absolutely necessary for the formation of bones. But that showed a lamentable want of chemical knowledge, because the lime required in food does not come from the water, but from the solid particles of food taken, and I do not think that the lime in water has any influence on the process of animal nutrition." Sir Lyon went on to add that "the water consumed in the mountainous districts of Scotland is soft water, and Highlanders are not generally supposed to be deficient in bone or muscle." On this same point, Dr. Angus Smith, formerly Chief Inspector of Alkali Works, stated before the same Royal Commission that he thought "that the tallest people in Great Britain are to be met with in soft-water districts, for instance, in Cumberland, and probably Aberdeen." The tallest people of all were found in Aberdeen, "which is a very soft-water district." Soft water is, in short, pure water, so far as lime is concerned; and both in sickness and in health, and, indeed, for all the ordinary purposes for which water is required, it is much to be preferred to hard.

**Honest Anti-Vaccinators?** IMMEDIATELY after the receipt of Mr. W. Tebb's letter on vaccination, which we published last week, another letter was sent to us, signed by Mr. Jas. R. Williamson. We have not space for any more of these; and, besides, the anti-vaccination controversy is not interesting, except to a few self-admiring faddists, who have no scientific capacity for understanding the subject. One point only in Mr. Williamson's communication demands attention. Mr. Williamson throws down certain challenges to Mr. Ernest Hart, editor of the *British Medical Journal*. He states that Mr. Hart has been repeatedly asked to give evidence before the Royal Commission on Vaccination, but has not done so. This is a categorical statement. We have communicated with Mr. Ernest Hart, and have received from him a statement equally categorical, but which affirms the direct contrary to be the truth. Mr. Hart says: "It is entirely untrue that I have been asked by the Royal Commission on Vaccination to give evidence before them; and it is, of course, alike untrue that I have refused to do so. All the evidence in my possession is based upon authentic documents, every one of which are also in the possession of the Royal Commission, and which they are perfectly able to interpret themselves. I have already stated this publicly, and Mr. Williamson's repeated mis-statements on the subject are, therefore, all the more inexcusable." Now, what is to be done with a controversialist like Mr. Williamson? He is as irrepressible as a "Jack-in-the-Box" with the lid off, and apparently just about as responsible. Argument is out of the question. One might as well argue with a crocodile who has got a black baby in his mouth. Science and sense are alike agreed that the anti-vaccinators should be left severely alone.

